# Household and individual level risk factors associated with declining malaria incidence in Meghalaya, India: implications for malaria elimination in low-endemic settings

**DOI:** 10.1186/s12936-021-03982-x

**Published:** 2021-12-11

**Authors:** Rajiv Sarkar, Anne Kessler, Bandapkupar Mawkhlieng, Steven A. Sullivan, Mark L. Wilson, Jane M. Carlton, Sandra Albert

**Affiliations:** 1 Indian Institute of Public Health - Shillong, Shillong, Meghalaya 793001 India; 2 Martin Luther Christian University, Shillong, Meghalaya 793006 India; 3 Center for Genomics and Systems Biology, Department of Biology, New York University, New York, NY 10003 USA; 4 Department of Epidemiology, School of Public Health, University of Michigan, Ann Arbor, MI 48109 USA; 5 Department of Epidemiology, School of Global Public Health, New York University, New York, NY 10003 USA

**Keywords:** Asymptomatic infection, Transmission intensity, Malaria elimination, Northeast India

## Abstract

**Background:**

A detailed analysis of household and individual level *Plasmodium* infection patterns in two low-endemic districts of Meghalaya was undertaken to better understand the epidemiology of malaria in northeast India.

**Methods:**

Socio-demographic and behavioural information from residents (aged 1–69 years) of households were collected through pre-tested, questionnaire conducted in 2018 and 2019. Blood samples collected from participants were tested for *Plasmodium falciparum* and/or *Plasmodium vivax* infection using rapid diagnostic test, microscopy and PCR. Plasma samples from a subset of participants were analysed for antibodies against thirteen *P. falciparum* and four *P. vivax* antigens. Associations between household and individual level risk factors, and *Plasmodium* infections were evaluated using multilevel logistic regression models.

**Results:**

A total of 2753 individuals from 827 households were enrolled in 2018, and 834 individuals from 222 households were enrolled in 2019. Of them, 33 (1.2%) were positive by PCR for *P. falciparum* in 2018 and none were positive for *P. vivax.* In 2019, no PCR-positive individuals were detected. All, but one, infections were asymptomatic; all 33 infections were sub-microscopic. Reported history of malaria in the past 12 months (OR = 8.84) and history of travel in the past 14 days (OR = 10.06) were significantly associated with *Plasmodium* infection. A significant trend of increased seropositivity with age was noted for all 17 antigens. Although adults (≥ 18 years) consistently had the highest seropositivity rates, a sizeable proportion of under-five children were also found to be seropositive. Almost all individuals (99.4%) reported sleeping under an insecticide-treated bed-net, and household indoor residual spray coverage in the 12 months preceding the survey was low (23%). Most participants correctly identified common signs and symptoms of malaria, i.e., fever (96.4%), headache (71.2%), chills (83.2%) and body-ache (61.8%). Almost all participants (94.3%) used government-provided services for treatment of malaria.

**Conclusion:**

This study explored the epidemiology of malaria in two communities in Meghalaya, India, in the context of declining transmission. The presence of widespread asymptomatic infections and seropositivity among under-five children suggest that low-level *Plasmodium* transmission persists in this region. Implications of the study findings for malaria elimination efforts in low-transmission settings are discussed.

## Background

Malaria continues to be a major public health problem globally with an estimated 229 million cases reported from 87 endemic countries [1]. India has the world's largest population at risk of malaria, with an estimated 162.5 million people living in high-transmission areas [2, 3]. Despite this, India has achieved a steady decline in the annual incidence of malaria from around 20 million in 2000 to 6 million in 2019, i.e., an absolute reduction of 73% [1].

In 2016, India launched the National Framework for Malaria Elimination with the ambitious goals of eliminating malaria from the country by 2030, maintaining malaria free status, and preventing reintroduction of infection in areas where transmission interruption has been achieved [4]. To achieve these goals, a five-year National Strategic Plan for Malaria Elimination was also launched in 2017 [5]. However, socio-cultural and behavioural beliefs and practices, undetected transmission from asymptomatic individuals, importation of infection from endemic areas, poor disease surveillance, resistance to antimalarial drugs and insecticides, and healthcare delivery and access issues may adversely impact the elimination efforts [6–8].

Even though the northeast region (NER) comprises about 4% of India’s population, it accounted for around 15% of the country's *Plasmodium falciparum* cases and 22% of the malaria deaths reported in 2019 [9]. Malaria incidence over the past decade has declined more slowly in the NER relative to the rest of the subcontinent [2]. This lag in declining incidence is partly due to the unique ecological and socio-cultural conditions of the NER, inhospitable terrain, poor road conditions and inadequate healthcare infrastructure, all of which may have contributed to the relatively high malaria incidence, until recently [3]. This is particularly true in the state of Meghalaya where incidence increased from 2012 to 2015, and only began to decline after 2016 [10, 11]. The reasons for the observed delayed decline in malaria incidence in Meghalaya are unclear but could be related to the first wide-spread distribution of long-lasting insecticidal nets (LLINs) in the endemic communities not occurring until 2016 [11].

Information on malaria patterns in Meghalaya is limited to unpublished government reports and a recent cross-sectional survey in two districts [11]. The later characterised village-level prevalence of *Plasmodium* infection during 2018–2019 in 21 villages, summarised village-level risk factors for infection, and identified 13 *Anopheles* mosquito species as potential vectors in a subset of villages [11]. To further understand the epidemiology of malaria in Meghalaya, the study presented here undertook detailed analysis of household and individual level *Plasmodium* infection patterns in the same two low-endemic communities in 2018–2019, evaluated social and behavioural risk factors for infection, and explored patterns of *Plasmodium* antigen-specific antibodies in the population.

## Methods

### Study area and population

**Fig. 1:**
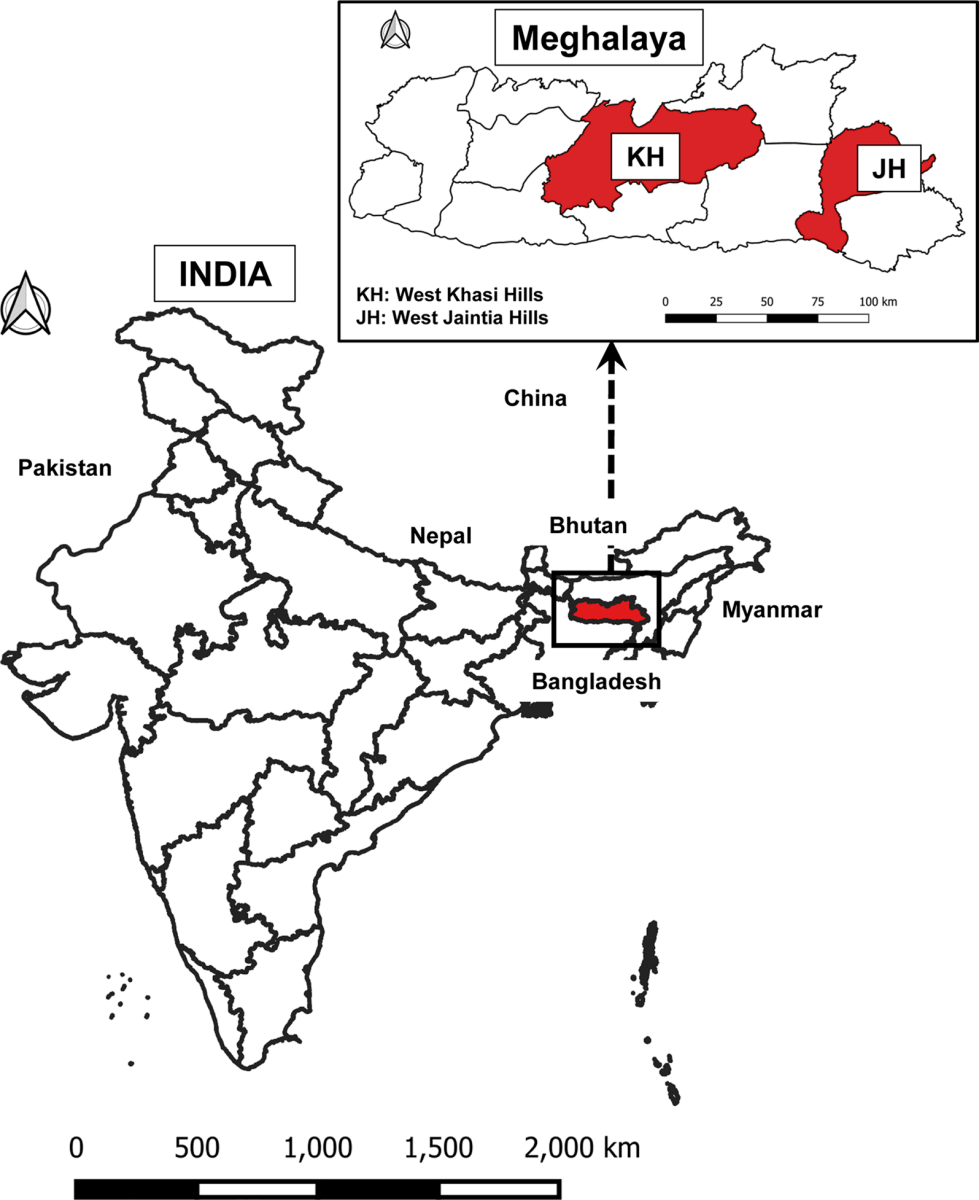
Map showing location of the study area

This study was conducted in two districts of Meghalaya: West Khasi Hills (KH) and West Jaintia Hills (JH). Meghalaya is a hilly and mountainous state in northeast India, located between Assam and Bangladesh (Fig. 1), with a population of about 3 million, mostly indigenous [12]. More than 75% of Meghalaya is forested [13]. The economy is predominantly agrarian; mixed farming (growing crops together with raising livestock) is a common practice [14]. The climate is the wettest in India especially during the May–September rainy season. With 23–28 °C temperatures and high relative humidity (> 70%) the conditions are conducive for mosquito breeding and perennial transmission. Between 2012 and 2015, the number of malaria cases and deaths in Meghalaya increased steadily, but seems to have generally declined from 2016 [10], although the rate of decline varied between and within districts [11].

### Data collected during community surveys

Socio-demographic and behavioural information was collected from residents (aged 1–69 years) of households in the JH and KH from April to November 2018 and again from May to September 2019. Details of the sampling design and methods have been described elsewhere [11]. Briefly, a total of 21 villages were selected for the study based on reported prior *Plasmodium* infections (both high and low prevalence), representation across primary health centres (PHC) and health sub-centres in the study area and location. A random sample of households from each village (selected based on probability proportion to size technique) were consented and enrolled. A household survey was administered to one resident of each participating household, usually the ‘head of household’ that included questions on family size, source of water supply, building materials for roofs and walls, presence of electricity, toilets, animals and malaria prevention methods (insecticide-treated nets [ITN], repellents, coils, DDT spraying). In addition, the household members were administered individual surveys during which they were asked mostly close-ended questions in their local language (Khasi, Pnar) to obtain information on age, gender, education, occupation, knowledge of malaria, health history (malaria episodes [preceding year], fever episodes [preceding 48 h]), travel history (past two weeks) and malaria prevention methods. Malaria risk behaviours and practices were also surveyed. Parents or caretakers responded on behalf of their children.

### Blood sample collection and *Plasmodium* detection

Details of the blood sample collection and *Plasmodium* detection methods have been described elsewhere [11]. Briefly, a small blood volume was taken by finger prick, and point-of-care detection of *P. falciparum* and/or *P. vivax* infections was determined by a bivalent rapid diagnostic test (RDT; FalciVax) and an ultra-sensitive RDT (Abbott Alere). Blood smears were also collected, fixed in methanol and Giemsa-stained prior to qualitative and quantitative evaluation by light microscopy. From the small blood volume, blood components were separated by centrifugation into plasma and a red blood cell (RBC) pellet and stored at -80 °C until assayed.

Species-specific *Plasmodium* infections were detected by PCR amplification of concentrated DNA extracted from microvette RBC pellets using a single-step PCR targeting Pfr364 (for *P. falciparum*) and Pvr47 (for *P. vivax*) genomes, respectively [15], as previously described [11]. A positive *Plasmodium* infection was indicated based upon RDT results and/or PCR results; no infections were detected by microscopy.

### Antibody quantification by Luminex MAGPIX

Seventeen recombinant *P. falciparum* and *P. vivax* proteins and/or peptides were coupled to unique Luminex Magplex magnetic microspheres and used in a multiplexed, bead-based assay to quantify host IgG antibodies as previously described [16]. Plasma samples from a total of 264 study participants were assayed. The samples were selected through a stratified random sampling technique, considering village as the strata; approximately 10% of the plasma samples collected in each village were tested. Plasma isolated from the small blood volumes collected by finger prick from the participants were prepared at 1/200 in a buffer of PBS, 0.05% Tween, 0.5% BSA, 0.02% sodium azide, 0.1% casein, 0.5% PVA, 0.5% PVP, and *E. coli* extract. All samples were assayed singularly with positive controls, naïve sample pools, and blanks run in duplicate or triplicate according to standardized procedures [17]. Plates were analysed on a Luminex MAGPIX, and xPONENT software was used for data acquisition (Luminex Corp., Austin, TX).

### Statistical analysis

Data were collected and managed using REDCap electronic data capture tools [18, 19] hosted at New York University and analysed using Stata software, version 14.2 for Windows (StataCorp, College Station, Texas). Data analyses were conducted using a complete-case approach, whereby participants with missing data for relevant variables were excluded. Summary statistics for continuous variables are presented as mean and standard deviation (SD) or median and interquartile range (IQR, 25th–75th percentile), depending on the distribution of data and as numbers and percentages (%) for categorical variables. The prevalence of *Plasmodium* infection, along with the 95% confidence interval (CI), was calculated using the Taylor linearized method that accounts for the clustered data-structure [20]. Associations between household and individual level socio-demographic, environmental and behavioural risk factors, and *Plasmodium* infections were evaluated using multilevel logistic regression models with exchangeable correlation matrix, considering village and household as grouping variables and reported as odds ratios (OR) with 95% CIs.

### Antibody data analysis

Net mean fluorescence intensity (MFI) (net MFI_Ag_ = raw MFI_Ag_ − background MFI_Ag_) where background MFI_Ag_ is the mean MFI of a given antigen in the blank wells was calculated for each antigen in each sample assayed. The seropositive threshold for each antigen was defined as mean net MFI_negative pool_ plus three standard deviations. The number and proportion of individuals seropositive for each antigen was tabulated for three age categories: children (1–7 years), adolescents (8–17 years), and adults (≥ 18 years). Differences in the proportion of seropositive individuals across the three age categories was determined by using the chi-square test for trend. The net MFI_Ag_ values from seropositive individuals were normalised with respect to the corresponding seropositive threshold value to generate a relative MFI value for comparison of response magnitudes (e.g., normalised relative values greater than one were considered seropositive for IgG to the respective antigen). The median (IQR) relative MFI values were determined for each antigen across all age groups, and differences in the relative magnitude of response across age groups was tested using a nonparametric test for trend across ordered groups [21].

### Ethical approval

Permission to undertake the study was obtained from the Institutional Review Board at New York University, New York, NY, USA and the University Research Ethics Committee of Martin Luther Christian University, Shillong, Meghalaya, India. Written informed consent was obtained from all adult participants (≥ 18 years of age). Assent was obtained for participants aged 7–17 years, in addition to the parental consent.

## Results

**Fig. 2:**
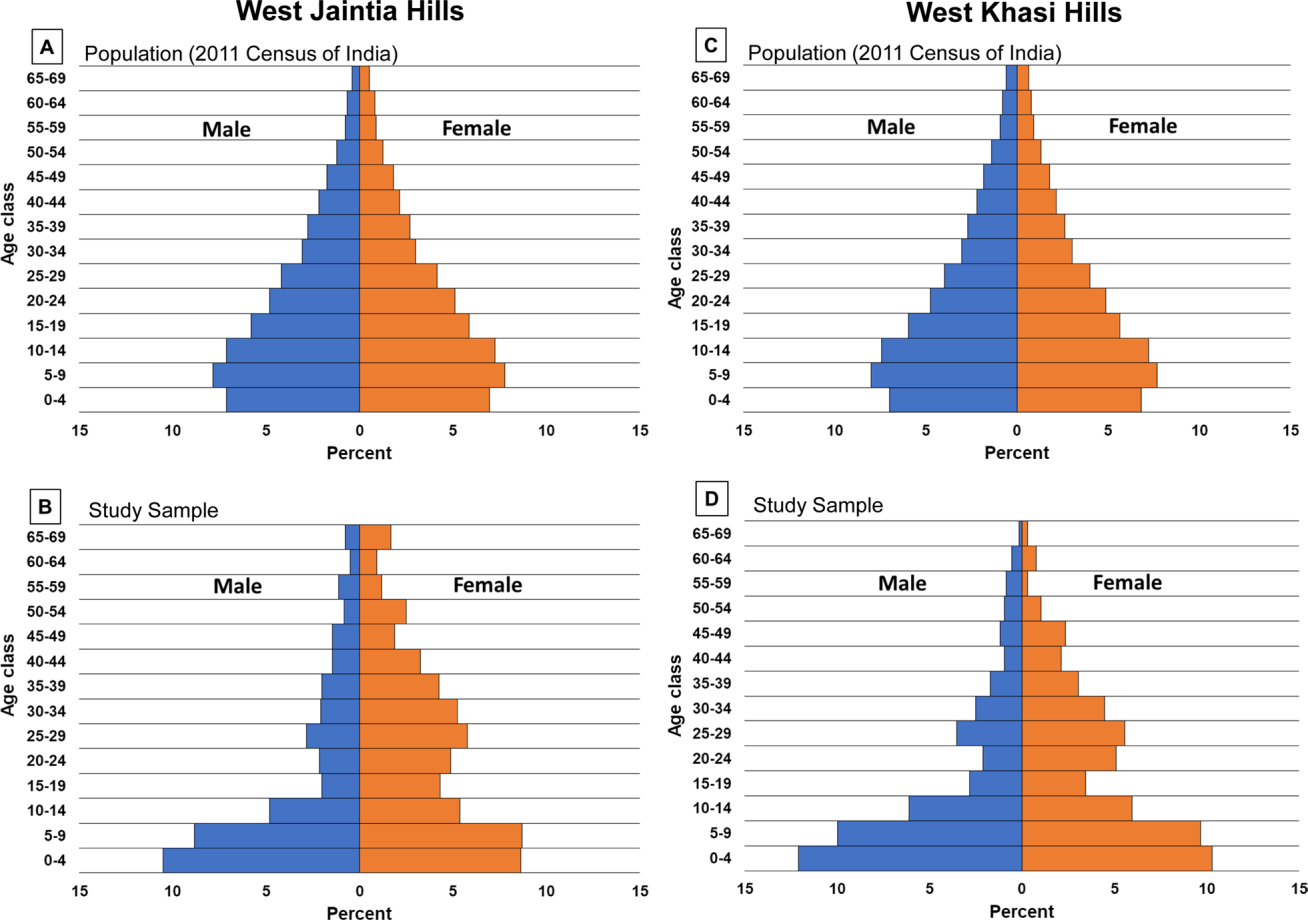
Population pyramid showing the age and gender distribution of: **A** Jaintia Hills district population ages 1–69 years as per the 2011 Census of India; **B** sampled population in the study villages from West Jaintia Hills district; **C** West Khasi Hills district population ages 1–69 years as per the 2011 Census of India; and **D** sampled population in the study villages from West Khasi Hills district

From 21 villages surveyed in JH (N = 9) and KH (N = 12) that represented 9306 residents from 1688 households, a total of 3017 (32.4%) individuals were approached for participation; 2753 individuals (29.6%) living in 820 households (48.6%) were enrolled in the study during 2018. In 2019, 222 households (13.2%) and 834 people (9%) from these villages were enrolled. The age and gender distribution of the total population of the two districts and that of the study participants is presented in Fig. 2. Compared to the age and gender distribution of the population at the district-level, the study participants had greater representation of children and female participants.

Blood from microvette samples during 2018 was available for testing from 1463 of the 1467 (99.7%) participants in JH, and 1234 of the 1286 (98.3%) KH participants. In 2018, 33 of 2697 study participants (1.2%, 95% CI 0.5–3.2%) were positive by PCR for *P. falciparum*; none of the participants tested positive for *P. vivax*. One (3%) of the 33 PCR-positive samples was also positive by RDT (both bivalent and ultra-sensitive). The prevalence of *P. falciparum* infection was similar in JH (1.1%, 95% CI 0.2–7.2%) and KH (1.4%, 95% CI 0.5–3.8%). In 2019, none of 834 blood samples (0%) were positive for either *Plasmodium* species, including samples from 18 PCR-positive individuals from the 2018 survey. For this reason, analysis of risk factors for *P. falciparum* was undertaken solely for 2018.

### Characteristics of the 2018 study population

#### Household level characteristics

The household level characteristics, aggregated by village, have been described elsewhere [11]. Briefly, the average (SD) household comprised of 5.6 (2.1) individuals. Brick (31.1%) was the commonest building material for the side walls, while the roofs were commonly made of tin (89.4%). More than two-thirds (68.1%) of the participating households had electricity and three-fourths (77.7%) had piped water supplied through indoor plumbing. A functional toilet was available in 88.2% of households, and almost all households (98.8%) with a functional toilet reported it being used all the time by household members. More than half (60.7%) of the respondent households had access to a mobile phone, and those with access reported mostly using it (84.7%) during the time of illness. More households in JH vs. KH had mobile phones (73.1% vs. 44.2%) and reported using them during sickness (62.6% vs. 36.4%). Animals were present in more than half (59.3%) of households, including poultry (52.0%) and pigs (27.7%); more than a third (38.3%) reported keeping animals inside the house.

#### Individual level characteristics

The mean (SD) age of participants was 20.8 (17.2) years. Slightly more females (56.5%) than males participated in the study. Of the adult participants (≥ 18 years), a majority (47.6%) had less than primary education (no formal education or completed preschool/kindergarten). Most participants were either students (35.8%) or engaged in agriculture-related activity (27.5%). Malaria diagnosis within the past 12 months was reported by only 3.6% of the participants; 2% of participants reported a history of travel outside of the village in the last 14 days. Around 5% of the participants reported staying in the field for one or more nights, with a mean (SD) of 6.9 (3.0) days. A large majority (86.2%) reported using ITNs while staying in the field. Most participants had knowledge of the common signs and symptoms of malaria, i.e., fever (96.4%), headache (71.2%), chills (83.2%) and body-ache (61.8%). Almost everyone (94.3%) preferred to seek treatment from government healthcare facility or community health worker (Accredited Social Health Activist or ASHA), if diagnosed with malaria.

The individual level socio-demographic characteristics of the study participants are presented in Table 1. The age and gender distribution of participants were similar between the two study areas. More adult participants in JH (55.7%) had below primary education than in KH (37%). A greater proportion of participants in KH reported being diagnosed with malaria in the past 12 months (6.5% vs 1.1%). On the other hand, a greater proportion of participants in JH reported staying overnight in the field for one or more days (7.9% vs 1.7%). Also, participants from JH (7.3 [3.0]) tended to stay longer in the field than those in KH (4.8 [1.9]), although the proportion of participants using ITNs while staying in the field was greater (94%) in JH than in KH (45.5%). The majority of participants in both study areas was correctly able to recall the signs and symptoms of malaria, although a larger proportion of respondents from JH (99.8%), as opposed to KH (88.1%), preferred to seek treatment from government healthcare facility or the ASHA if diagnosed with malaria.

**Table 1:** Individual level social and demographic characteristics of study participants in two districts of Meghalaya state, India

Characteristic	West Jaintia Hills (N = 1467)	West Khasi Hills (N = 1286)
Age (years)*	22.2 (18.1)	19.2 (16.0)
Gender		
Female	855 (58.3%)	697 (54.4%)
Male	612 (41.7%)	585 (45.6%)
Highest education of participants aged ≥ 18 years^		
No formal education	338 (45.7%)	160 (28.6%)
Below primary (preschool/kindergarten)	74 (10.0%)	47 (8.4%)
Primary (class V)	221 (29.9%)	104 (18.6%)
Middle (class VIII)	48 (6.5%)	125 (22.3%)
Secondary/matric (class X)	31 (4.2%)	64 (11.4%)
Higher secondary (class XII)	13 (1.8%)	34 (6.1%)
Graduate	14 (1.9%)	25 (4.5%)
Post graduate	0 (0%)	1 (0.2%)
Diploma	1 (0.1%)	0 (0%)
Occupation		
Cultivator	349 (23.8%)	269 (20.9%)
Agricultural labourer	124 (8.5%)	14 (1.1%)
Daily wage/labour	82 (5.6%)	76 (5.9%)
Salaried service	28 (1.9%)	36 (2.8%)
Self-employed/trade	16 (1.1%)	15 (1.2%)
Housewife	127 (8.7%)	125 (9.7%)
Student	473 (32.3%)	512 (39.8%)
Child, not schooling	61 (4.2%)	28 (2.2%)
None	206 (14.1%)	205 (15.9%)
Other	0 (0%)	6 (0.5%)
Self-reported malaria in past 12 months	16 (1.1%)	83 (6.5%)
History of travel in last 14 days	31 (2.1%)	25 (1.9%)
Staying in field for one or more night		
Yes	116 (7.9%)	22 (1.7%)
No	684 (46.7%)	663 (51.6%)
Not applicable	665 (45.4%)	600 (46.7%)
Number of nights stayed in the field*	7.3 (3.0)	4.8 (1.9)
Taking ITN when staying in the field	109 (94.0%)	10 (45.5%)
Knowledge of signs and symptoms of malaria		
Fever	88 (98.9%)	315 (96.3%)
Headache	84 (94.4%)	213 (65.3%)
Chill	68 (76.4%)	249 (85.2%)
Body-ache	49 (55.1%)	187 (63.8%)
Preferred healthcare service provider for malaria		
Government healthcare facility	1464 (99.8%)	1133 (88.1%)
Private healthcare facility	3 (0.2%)	147 (11.4%)
Traditional healer/home management	0 (0%)	4 (0.3%)
Don’t know/other facilities	0 (0%)	2 (0.2%)

*Mean (SD)

^Only the 740 adult (aged ≥ 18 years) participants from JH and 540 adult participants from KH were included in the analysis

### Risk factors of malaria infection

The household and individual level risk factors for *Plasmodium* infections were assessed separately for JH and KH. A total of 2697 individuals from 819 households for whom a PCR-test result for *Plasmodium* infection was available were included in the analysis (1463 from 468 households in JH, and 1234 from 351 households in KH).

#### Household level risk factors for *Plasmodium* infection

A variety of potential household characteristics and household level behaviours were analysed as risk for *Plasmodium* infection in individual household members (Table 2). These included presence of electricity and mobile phones, house roof and wall construction materials, presence of animals, anti-mosquito prevention measures, including indoor residual spraying. None of the household level factors were found to significantly predict the risk of *Plasmodium* infection in the study population.

**Table 2:** Household level risk factors for *Plasmodium* infection in two districts of Meghalaya state, India

	West Jaintia Hills (N = 468 households)		West Khasi Hills (N = 351 households)	
	OR (95% CI)*	*P*-value*	OR (95% CI)*	*P*-value*
Presence of electricity	1.73 (0.26–11.69)	0.573	1.89 (0.40–8.99)	0.424
Presence of mobile phone	0.53 (0.11–2.57)	0.432	2.21 (0.72–6.81)	0.165
Walls made of mud/thatch/wood^	0.38 (0.03–4.86)	0.456	0.73 (0.25–2.13)	0.563
Roof made of thatch/tile^¶^	–^‡^	–^‡^	2.06 (0.41–10.40)	0.383
Presence of animals				
Any animal	1.05 (0.25–4.34)	0.949	1.89 (0.65–5.47)	0.241
Pigs	1.39 (0.32–6.03)	0.661	0.80 (0.20–3.16)	0.750
Poultry	2.06 (0.49–8.64)	0.323	1.10 (0.38–3.18)	0.857
Buffalo/cow	–^‡^	–^‡^	3.67 (0.37–37.00)	0.269
Animals living inside the house	0.54 (0.12–2.41)	0.421	1.33 (0.46–3.83)	0.600
Mosquito always present	0.92 (0.22–3.92)	0.913	2.23 (0.72–6.91)	0.163
Presence of mosquito repellent coils in household	0.55 (0.14–2.16)	0.390	2.43 (0.80–7.34)	0.115
Presence of mosquito repellent mat tablets in household	0.50 (0.08–3.07)	0.454	0.92 (0.11–7.79)	0.938
Presence of mosquito repellent vaporizers in household	2.52 (0.44–14.59)	0.301	0.94 (0.11–8.28)	0.955
Method for reducing mosquito burden				
Insecticide spray	–^‡^	–^‡^	3.48 (0.96–12.61)	0.058
Burning neem leaves or cow dung	–^‡^	–^‡^	1.00 (0.10–10.04)	0.997
Clear bushes around the house	–^‡^	–^‡^	0.62 (0.07–5.80)	0.671
Clear stagnant water pools	–^‡^	–^‡^	1.35 (0.16–11.36)	0.781
Keep windows/doors closed in evening	–^‡^	–^‡^	0.35 (0.06–1.94)	0.228
Screening of windows	–^‡^	–^‡^	1.48 (0.36–5.94)	0.582
Presence of chalk markings in house indicating DDT spraying	–^‡^	–^‡^	2.23 (0.48–10.30)	0.303

*The OR and 95% CIs were obtained from multilevel logistic regression models, with *Plasmodium* positivity at the individual level as the outcome variable, and household and individual level factors as the exposure variables

^Reference category: wall made of brick/concrete/stone

^¶^Reference category: roof made of concrete/tin

^‡^Odds ratio (95% CI) and *P*-value could not be estimated because of separation due to small number of participants with *Plasmodium* infection

#### Individual level risk factors for *Plasmodium* infection

The individual level risk factors for *Plasmodium* infection were also analysed (Table 3). A reported history of malaria in the past 12 months (OR = 8.84, *P* = 0.046) was significantly associated with *Plasmodium* infection in JH and history of travel in the past 14 days (OR = 10.06, *P* = 0.008) was significantly associated with *Plasmodium* infection in KH. None of the other factors were significantly associated with risk of *Plasmodium* infection in either of the two study districts.

**Table 3:** Individual level risk factors for *Plasmodium* infection of study participants in two districts of Meghalaya state, India

	West Jaintia Hills (N = 1463 Individuals)		West Khasi Hills (N = 1234 Individuals)	
	OR (95% CI)*	*P*-value*	OR (95% CI)*	*P*-value*
Age (in years)	1.01 (0.98–1.04)	0.515	0.99 (0.96–1.02)	0.693
Male sex	0.45 (0.13–1.56)	0.207	0.66 (0.23–1.84)	0.424
Below primary education^	0.65 (0.20–2.12)	0.480	0.68 (0.24–1.89)	0.456
Occupation: Cultivator/Agricultural labourer	0.89 (0.24–3.27)	0.861	0.87 (0.27–2.86)	0.824
Reported history of malaria in past 12 months	**8.84 (1.03–75.15)**	**0.046**	–^‡^	–^‡^
Travelled in past 14 days	–^‡^	–^‡^	**10.06 (1.85–55.68)**	**0.008**
Stayed in field for one or more night(s)	1.38 (0.11–17.06)	0.800	4.60 (0.47–45.42)	0.192
Knowledge of malaria signs/symptoms	0.46 (0.04–4.83)	0.515	0.51 (0.11–2.32)	0.381
Leaves house at night to use toilet	1.05 (0.25–4.45)	0.944	2.08 (0.73–5.89)	0.057
Uses bed net every night	0.90 (0.22–3.72)	0.889	1.02 (0.26–3.96)	0.978
Never cover arms/legs against mosquitoes	1.05 (0.29–3.84)	0.943	0.39 (0.13–1.22)	0.106
Uses insecticidal cream against mosquitoes	–^‡^	–^‡^	0.68 (0.08–5.83)	0.726
Other products used to prevent bites				
Mosquito repellent coil	0.66 (0.17–2.60)	0.549	1.68 (0.56–4.99)	0.351
Mosquito repellent mat tablet	0.52 (0.09–3.16)	0.477	–^‡^	–^‡^
Mosquito repellent vaporizer	2.95 (0.50–17.51)	0.233	0.71 (0.08–6.14)	0.759
Burn other materials	–^‡^	–^‡^	0.92 (0.09–9.30)	0.946
Experienced fever in the past 48 h	–^‡^	–^‡^	1.17 (0.14–9.91)	0.884

*The OR and 95% CIs were obtained from multilevel logistic regression models, with *Plasmodium* positivity at the individual level as the outcome variable, and household and individual level factors as the exposure variables

^Individuals with no formal education or completed preschool/ kindergarten

^‡^Odds ratio (95% CI) and *P*-value could not be estimated because of separation due to small number of participants with *Plasmodium* infection

### Presence and relative magnitude of anti-*P. falciparum* and anti-*P. vivax* antibodies in a subset of study participants

The number and proportion of seropositive individuals in each age category are listed by antigen/antibody in Table 4. For each antigen assayed, two or more individuals in each age category were found to be seropositive. The proportion of seropositive individuals by age group was significantly different for all 17 antigens, and the greatest proportion of seropositive individuals was consistently observed in the adult age category.

**Table 4:** Presence and relative magnitude of antibodies to *P. falciparum* and *P. vivax* antigens by age in a subset of 264 participants

Antigen/ Antibody	Number of seropositive individuals n (% of age category)				Relative magnitude of response* Relative MFI value (IQR)			
	Age (1–7 years) (N = 77)	Age (8–17 years) (N = 72)	Age (≥ 18 years) (N = 115)	*P*-value^	Age (1–7 years)	Age (8–17 years)	Age (≥ 18 years)	*P*-value^¶^
PfAMA1	30 (39.0%)	31 (43.1%)	98 (85.2%)	< 0.001	1.21 (1.10–1.57)	1.52 (1.24–1.69)	4.83 (1.92–13.64)	< 0.001
PfEBA140	13 (16.9%)	13 (18.1%)	63 (54.8%)	< 0.001	2.40 (1.53–3.38)	1.86 (1.50–2.89)	2.22 (1.52–4.60)	0.720
PfEBA175	9 (11.7%)	12 (16.7%)	82 (71.3%)	< 0.001	1.09 (7.35–1.79)	1.86 (1.19–2.43)	3.74 (2.15–7.45)	< 0.001
PfEBA181	6 (7.8%)	15 (20.8%)	69 (60.0%)	< 0.001	1.46 (1.25–2.75)	1.35 (1.19–2.90)	3.65 (1.97–6.10)	0.001
PfEtramp5.Ag1	12 (15.6%)	16 (22.2%)	67 (58.3%)	< 0.001	1.34 (1.20–1.94)	1.49 (1.12–1.69)	2.25 (1.48–4.20)	0.001
PfGlurp.R2	5 (6.5%)	11 (15.3%)	60 (69.6%)	< 0.001	1.77 (1.42–2.17)	1.38 (1.08–3.37)	4.72 (1.94–10.78)	0.007
PfHSP40.Ag1	9 (11.7%)	9 (12.5%)	57 (49.6%)	< 0.001	1.43 (1.15–2.14)	2.05 (1.11–2.15)	1.95 (1.43–2.99)	0.077
PfMSP1_19_	4 (5.2%)	6 (8.3%)	91 (79.1%)	< 0.001	1.50 (1.12–2.29)	1.13 (1.04–1.25)	5.45 (2.70–16.35)	< 0.001
PfMSP2_Ch150	3 (3.9%)	4 (5.6%)	45 (39.1%)	< 0.001	1.01, 1.02, 1.39	1.11 (1.09–1.88)	1.49 (1.22–2.93)	0.058
PfMSP2_Dd2	23 (29.9%)	10 (13.9%)	68 (59.1%)	0.002	2.70 (1.36–16.19)	1.91 (1.20–2.48)	3.47 (1.92–7.71)	0.020
PfRh4.2	22 (28.6%)	20 (27.8%)	74 (64.4%)	0.001	2.62 (1.47–4.66)	2.34 (1.72–3.16)	2.21 (1.44–4.02)	0.678
PfRh2 2030	20 (26.0%)	16 (22.2%)	87 (75.7%)	< 0.001	1.35 (1.14–1.86)	1.62 (1.25–2.35)	4.74 (1.99–9.80)	< 0.001
PfRh5	7 (9.1%)	14 (19.4%)	59 (51.3%)	< 0.001	1.15 (1.06–2.72)	1.63 (1.14–2.11)	2.29 (1.40–3.08)	0.011
PvAMA1	2 (2.6%)	3 (4.2%)	73 (63.5%)	< 0.001	1.19, 1.89	2.90 (1.25–3.28)	5.04 (2.32–14.26)	0.020
PvMSP10	28 (38.4%)	24 (33.3%)	94 (81.7%)	< 0.001	1.57 (1.29–2.11)	1.43 (1.19–2.93)	2.47 (1.45–4.06)	0.002
PvMSP1_19_	2 (2.6%)	4 (5.6%)	58 (50.4%)	< 0.001	1.36, 2.14	1.61 (1.43–1.93)	2.18 (1.50–5.10)	0.218
PvMSP8	22 (28.6%)	28 (38.9%)	92 (80.0%)	< 0.001	1.83 (1.16–2.60)	1.75 (1.39–3.03)	2.02 (1.40–3.31)	0.164

*Only seropositive individuals were included in the analysis of each antigen

^Chi-square test for trend for difference in the proportion of seropositive individuals across age categories ( 1–7 years; 8–17 years; ≥ 18 years)

^¶^Non-parametric test for trend for the difference in the magnitude of response in seropositive individuals across age categories (1–7 years; 8–17 years; ≥ 18 years)

The relative magnitude of antibody detected was significantly different across age groups for eight of the 13 *P. falciparum* antigens assayed: PfAMA1 (*P* < 0.001), PfEBA175 (*P* < 0.001), PfEBA181 (*P* = 0.001), PfEtramp5.Ag1 (*P* = 0.001), PfGlurp.R2 (*P* = 0.007), PfMSP1_19_ (*P* < 0.001), PfRh2 2030 (*P* < 0.001), and PfRh5 (*P* = 0.011) (Table 4). The relative magnitude of antibody was greatest in adult individuals (≥18 years of age) for all eight of the aforementioned targets with the greatest relative MFIs observed for antibodies against PfMSP1_19_ (5.45), PfAMA1 (4.83), Rh2 2030 (4.74) and PfGlurp.R2 (4.72).

The relative magnitude of antibody detected was significantly different across age groups for two of the four *P. vivax* antigens, PvAMA1 (*P* = 0.020) and PvMSP10 (*P* = 0.002). Independent of statistical significance, the relative magnitude of antibody was greatest in the adult age category for all four *P. vivax* targets with the highest relative MFI observed for PvAMA1 (5.04).

### Reported malaria risk-reduction

#### Household level responses

Questions about presence of mosquitoes and methods for reducing household level human exposure indicated widespread mosquito abundance and extensive efforts to decrease contact (Table 5). Mosquitoes were always (45.4%) or sometimes (54.5%) present in essentially every household of both study districts. Nearly every household reported intentionally attempting to reduce mosquito abundance by clearing vegetation (98.2%) and removing stagnant water around dwellings (97.6%), as well as keeping windows and doors closed in the evening (97.7%). The ITNs were universally present (99.3%), numbering 2 to 3 per household, and were well-maintained by washing (88.8%). However, the average (SD) age of ITNs was 2.1 (0.5) years. More than two-third of the ITNs (78.6%) had holes due to wear and tear, most of which (96.8%) were repaired. Although roughly half of households (44.5%) reported sometimes burning insecticide coils indoors, interior walls were sprayed with residual insecticides (IRS) in less than half (46.3%) of KH houses and only 6.2% of dwellings in JH. The commonest reason cited for not spraying walls with the IRS was the spray team not having visited the household (68.2%), presence of a child (7.7%) and non-availability of a household member when the spray team visited the household (5.2%).

**Table 5:** Household level mosquito risk and prevention methods in 820 households from two districts of Meghalaya state, India

Characteristic	West Jaintia Hills (N = 468)	West Khasi Hills (N = 352)
Mosquitoes present in house		
Yes, always	263 (56.2%)	109 (31.0%)
Yes, sometimes	125 (26.7%)	102 (29.0%)
Yes, rainy season only	80 (17.1%)	140 (39.7%)
No	0 (0%)	1 (0.3%)
Methods used to reduce mosquitoes		
Clear bushes around the house	467 (99.8%)	337 (96.0%)
Clear stagnant water pools	466 (99.6%)	333 (94.8%)
Keeping windows/doors closed in evening	467 (99.8%)	334 (94.9%)
Screening of windows	10 (2.1%)	147 (42.0%)
Insecticide spray	2 (0.4%)	20 (5.7%)
Use larvicide in ponds	2 (0.4%)	3 (0.9%)
Burn other materials	3 (0.6%)	45 (12.8%)
ITN presence, condition, treatment		
Any ITNs present in house	466 (99.6%)	343 (98.9%)
Number of ITNs present: Median (IQR)*	2 (2–3)	3 (2–3)
Duration (years) of ITN use: Mean (SD)*	2.0 (0.2)	2.2 (0.8)
Washing of ITN by HH members*	463 (99.4%)	260 (74.9%)
Wash frequency: Median (IQR)^	4 (3–4)	2 (2–3)
Holes/defects in ITN*	400 (86.2%)	233 (68.3%)
Repair of holes/defects in ITN^¶^	392 (98.0%)	221 (94.9%)
DDT spraying by NVBDCP (HH marked)	25 (5.3%)	117 (49.4%)
IRS spraying of house (< 12 months)		
Yes	29 (6.2%)	163 (46.3%)
No	439 (93.8%)	181 (51.4%)
Don't know	0 (0%)	8 (2.3%)
Reason for not using IRS in house		
Spray team did not visit the household	391 (89.1%)	32 (17.7%)
Presence of children	14 (3.2%)	34 (18.8%)
Not at home at the time of spraying	10 (2.3%)	22 (12.2%)
Don't like the smell	16 (3.6%)	22 (12.2%)
Stains the walls	1 (0.2%)	10 (5.5%)
Dangerous for silk production	9 (2.1%)	0 (0%)
Mosquito repellent coils present in house	264 (56.4%)	101 (28.7%)
Time of day coils used in house^‡^		
Night time	162 (61.4%)	62 (61.4%)
Evening	157 (59.5%)	51 (50.5%)
Daytime	0 (0%)	0 (0%)
Frequency of mosquito repellent coil use^‡^		
Always	21 (8.0%)	25 (25.3%)
Sometimes	242 (92.0%)	72 (72.7%)
Rarely	0 (0%)	2 (2.0%)

*For the households reporting presence of ITN

^For the households reporting washing of ITN

^¶^For the households reporting holes/defects in ITN

^‡^For the households reporting presence of coils

#### Individual level responses

Individual level anti-mosquito protection was also widespread and appropriate (Table 6). Virtually all individuals (99.4%) reported sleeping under an ITN regularly (63.4%) or most of the time (29.7%). While roughly half of residents (43.1%) wore clothing to cover arms and legs to reduce mosquito bites, almost none (97.1%) used insecticidal creams to prevent bites. Evening activities (97.2%), including dinner (99.7%), almost always took place inside the house. Essentially everyone (99.8%) slept inside the house, although many (60.5%) reported leaving the house at night to use the toilet. Early morning activities were reported mostly (81.7%) to occur inside the house.

**Table 6:** Individual level mosquito risk and prevention methods among 2,753 study participants from two districts of Meghalaya state, India

Characteristic	West Jaintia Hills (N = 1467)	West Khasi Hills (N = 1286)
Generally, sleep under bed net at night	1467 (100%)	1270 (98.8%)
If yes, bed net treated with insecticide		
Yes	1457 (99.4%)	1245 (98.1%)
No	7 (0.5%)	24 (1.9%)
If yes, frequency of bed net use		
Every night	738 (50.3%)	998 (78.5%)
Most of the times	724 (49.4%)	90 (7.1%)
Sometimes	4 (0.3%)	36 (2.8%)
Rarely	0 (0%)	3 (0.2%)
Only in rainy season	1 (0.1%)	142 (11.2%)
Used bed net preceding night	1456 (99.3%)	1247 (97.2%)
Cover arms/legs to prevent bites		
Always	19 (1.3%)	169 (13.1%)
Sometimes	770 (52.5%)	229 (17.8%)
Rarely	6 (0.4%)	155 (12.1%)
Never	672 (45.8%)	733 (57.0%)
Use insecticidal creams to prevent bites		
Always	5 (0.3%)	11 (0.8%)
Sometimes	16 (1.1%)	25 (1.9%)
Rarely	4 (0.3%)	18 (1.4%)
Never	1442 (98.3%)	1232 (95.8%)
Other products used to prevent bites		
Mosquito repellent coil	806 (54.9%)	377 (29.3%)
Mosquito repellent mat tablet	105 (7.2%)	89 (6.9%)
Mosquito repellent vaporizer	51 (3.4%)	53 (4.1%)
Burn other materials	20 (1.4%)	158 (12.3%)
Dinner eaten inside the house	1467 (100%)	1284 (99.9%)
Location of activities before sleeping		
Inside the house	1402 (95.6%)	1270 (99.0%)
Outside the house	60 (4.1%)	11 (0.9%)
Depends on conditions	5 (0.3%)	2 (0.2%)
Sleeping location		
Inside the house	1465 (99.9%)	1280 (99.6%)
Outside the house	1 (0.1%)	2 (0.2%)
Depends on conditions	1 (0.1%)	3 (0.2%)
Leave house at night to use toilet	1089 (74.2%)	576 (44.9%)
Location of early morning activities*		
Inside the house	325 (72.5%)	691 (86.9%)
Outside the house	98 (21.9%)	89 (11.2%)
Location depending on season	24 (5.4%)	5 (0.6%)
Location changes every time	1 (0.2%)	11 (0.9%)

*Data available for 448 individuals in JH and 795 individuals in KH

## Discussion

This community-based cross-sectional study investigated the prevalence, and household and individual level risk factors of malaria to better understand the epidemiology of *Plasmodium* infection in the context of ongoing intensive malaria elimination effort in two districts of Meghalaya that only a few years ago had relatively high transmission [10]. A low prevalence of *Plasmodium* infection in the two communities in 2018 was determined, with malaria prevalence declining to essentially undetectable levels in 2019. Ninety-seven percent of the infected individuals had asymptomatic, sub-microscopic infection, which could only be detected through molecular (PCR-based) methods. A high prevalence of sub-microscopic infections was previously reported from malaria-endemic areas in India [22–24] and elsewhere [25–27]. As sub-microscopic carriers may contribute to sustained transmission of *Plasmodium* infection [28], the use of molecular diagnostic tools for detection of low-density infections has been recommended for malaria surveillance in low-endemic settings [29].

Plasma samples from a subset of the study participants were analysed for presence of antibodies against thirteen *P. falciparum* and four *P. vivax* antigens, chosen based upon prior classification as indicators of protection from clinical disease [30] or markers of cumulative exposure or recent infection [31]. The proportion of seropositive individuals, as well as the magnitude of serological response increased with age, which provides further evidence of the decrease in transmission in this area. A positive age-dependent seroconversion pattern has previously been reported from areas with low *Plasmodium* transmission intensity [32–35]. The presence of antibodies to several *Plasmodium* antigens in children under five years of age, however, can be considered as a marker of relatively recent exposure [36] and provides evidence of continued low-intensity transmission in the area that needs to be monitored.

A history of travel outside of the village within the past 14 days was associated with a higher risk of *Plasmodium* infection in this study. With increasing movement of people (locally, regionally, and globally), imported malaria from cross-border and regional human movement represents a major obstacle to malaria elimination [37, 38], especially in NE India [9] that shares vast international borders with five countries, including Bangladesh, Myanmar and Bhutan, all of which are malaria-endemic countries [1]. This highlights the need for strict vigilance of the border areas to prevent reintroduction of malaria in the post-elimination era.

A reported history of malaria in the past 12 months was associated with a higher risk of *P. falciparum* infection in this study. Recurrent episodes of malaria occurring in a small percentage of individuals have earlier been reported from endemic areas [39–41]. In a longitudinal study from Kenya, 21% of participants were found to contribute to 55% of the clinical malaria cases in that population [42]. Mathematical models have demonstrated an over-dispersion in the prevalence of malaria, which is generally considered to follow the ‘80/20’ rule, i.e., 80% of infections occur in 20% of the population [43, 44]. The reasons for this over-dispersion are not fully established and are attributed to a combination of host (such as human genetics and behaviour) [45–47] and environmental factors (such as distance from mosquito breeding area, wind pattern and exposure to infectious mosquitoes) [41, 45, 48].

Virtually every participant in the present study reported using ITNs. In a recent meta-analysis, ITN use was associated with 45% and 39% reduction in incidence of uncomplicated episodes of *P. falciparum* and *P. vivax* malaria, respectively [49]. In another meta-analysis study, ITNs were found to be more effective than untreated bed-nets regardless of insecticide resistance, although substantial heterogeneity between studies was noted [50]. Also, nearly every household reported that holes in ITNs were repaired. In a cohort study in Malawi with consistently high ITN usage, use of nets without holes conferred significantly greater protection than using nets with holes, despite moderate levels of insecticide resistance [51].

Indoor residual spray coverage in the study districts was low, with only 23% of the households reported having been sprayed in the preceding 12 months, much less than the World Health Organization target of > 85% IRS coverage for preventing malaria transmission [52]. Low levels of IRS coverage have been reported by other studies conducted in India [53, 54] and elsewhere [55], and is attributed to various factors such as lack of knowledge about IRS benefits, spray operators' behaviours, resident's reluctance to remove household items, and preference for using ITNs [54, 56, 57]. The present study found that the main reasons for low IRS coverage were that the spray team did not visit, an adult household member was not present when the spray team visited, refusal due to the presence of children or the dislike of the IRS smell. With increasing concern over reduced effectiveness of LLINs due to pyrethroid resistance [58–60], increasing the IRS coverage and acceptability through community participation [61] may be needed to sustain the gains in malaria elimination in endemic settings.

The lack of understanding of what causes malaria, the use of ineffective prevention methods, the belief that malaria cannot be prevented, and general reliance on traditional remedies have been cited as major barriers to malaria prevention and treatment [62]. In Meghalaya, most study participants were knowledgeable of malaria symptoms, regularly practiced appropriate malaria prevention, and sought treatment in a government healthcare facility. These findings are also consistent with the decrease in transmission and partly explain the difficulty in identifying risk factors associated with positive infection status.

Despite enrolling more than 2500 participants, the low prevalence of *Plasmodium*-positive individuals resulted in reduced statistical power to evaluate the risk factors for infection in this population, which is a limitation of this study. Future studies in low-transmission settings may consider including serological markers of recent infection as a tool to estimate the burden and transmission patterns of malaria in low-intensity settings [25, 63]. As with all community-based studies, the individuals participating in the cross-sectional survey may not be a representative sample of the study population (Fig. 2), possibly because of out-migration for employment or education. As migrants are considered to be at higher risk of malaria, especially if travelling to malaria-endemic areas [64, 65], the overall *Plasmodium* infection prevalence may have been underestimated in this study. Finally, detailed exposure history could not be obtained for participants with serology results, which limited the ability to differentiate between recent and long-term exposure to *Plasmodium* infection.

## Conclusion

This study provides important insights into the epidemiology of malaria in a low-transmission setting in India. The high proportion of asymptomatic *Plasmodium* infections and the presence of anti-*Plasmodium* antibodies detected in children under five years of age suggests “hidden” transmission in the region, although the relative contribution of asymptomatic and sub-microscopic individuals to parasite transmission and/or clinical malaria in low-transmission settings need to be explored further. The higher infection risk observed among those with a travel history in a region with extensive international borders, heterogeneous distribution of *Plasmodium* infections, abundant vector populations, and favourable environmental conditions [9, 11] altogether highlight the importance of constant vigilance to counter the threat of malaria resurgence from imported cases. Periodic serological surveys may be considered to monitor temporal trends in malaria transmission and to monitor progress towards elimination [32, 66]. Considering the widespread ITN usage among the study participants, monitoring for insecticide resistance should be undertaken to ensure continued effectiveness of vector-control methods.

## Data Availability

Data generated in this study are available through the open-access online resource for population-based epidemiological studies ClinEpiDB (https://clinepidb.org) at https://clinepidb.org/ce/app/record/dataset/DS_4670e06911.
